# Characterization and utilization of an international neurofibromatosis web-based, patient–entered registry: An observational study

**DOI:** 10.1371/journal.pone.0178639

**Published:** 2017-06-23

**Authors:** Mindell Seidlin, Robert Holzman, Pamela Knight, Bruce Korf, Vanessa Rangel Miller, David Viskochil, Annette Bakker, on behalf of the Children’s Tumor Foundation

**Affiliations:** 1 Children’s Tumor Foundation, New York, United States of America; 2 Department of Medicine, New York University School of Medicine, New York, United States of America; 3 Department of Genetics, University of Alabama at Birmingham, Birmingham, Alabama, United States of America; 4 Invitae, San Francisco, California, United States of America; 5 Department of Pediatrics, University of Utah School of Medicine, Salt Lake City, Utah, United States of America; Turun Yliopisto, FINLAND

## Abstract

The neurofibromatoses (neurofibromatosis type 1, neurofibromatosis type 2 and schwannomatosis) are rare disorders having clinical manifestations that vary greatly from patient to patient. The rarity and variability of these disorders has made it challenging for investigators to identify sufficient numbers of patients with particular clinical characteristics or specific germline mutations for participation in interventional studies. Similarly, because the natural history of all types of neurofibromatosis (NF) is variable and unique for each individual, it is difficult to identify meaningful clinical outcome measures for potential therapeutic interventions. In 2012, the Children’s Tumor Foundation created a web-based patient-entered database, the NF Registry, to inform patients of research opportunities for which they fit general eligibility criteria and enable patients to contact investigators who are seeking to enroll patients in approved trials. Registrants were recruited through CTF-affiliated NF clinics and conferences, through its website, and by word-of-mouth and social media. Following online consent, demographic information and details regarding manifestations of NF were solicited on the Registry website. Statistical analyses were performed on data from a cohort of 4680 registrants (the number of registrants as of October 9, 2015) who met diagnostic criteria for one of the 3 NF conditions. The analyses support our hypothesis that patient-reported symptom incidences in the NF Registry are congruent with published clinician-sourced data. Between April 26, 2013 and July 8, 2016, the registry has been useful to investigators in recruitment, particularly for observational trials, especially those for development of patient-reported outcomes.

## Introduction

The Children’s Tumor Foundation (CTF) is a 501(c)(3) medical foundation focused on research, education, advocacy and patient support in the neurofibromatoses including neurofibromatosis type 1 (NF1), neurofibromatosis type 2 (NF2) and schwannomatosis (SCHW). The neurofibromatoses are rare disorders with incidences of about 1 in 3000 live births for NF1, one in 30 000 for NF2 and one in 40 000 for SCHW. Each of these conditions may lead to multiple clinical manifestations, which vary greatly from patient to patient. Each may be inherited in an autosomal dominant pattern, or may occur *de novo*. Although there are many clinical trials ongoing, there are currently no disease-specific FDA-approved therapies for any of the complications of NF1, NF2 or SCHW.

The variability of manifestations in the neurofibromatoses results in difficulty in predicting the clinical course for any given individual. Although there have been efforts to identify genotype-phenotype correlations, the natural histories of the disorders and the relationship to genotypes are not fully characterized [1,2]. The rarity and variability of NF make it particularly challenging for investigators to identify sufficient numbers of patients with specific clinical characteristics or germline mutations for participation in interventional studies. Finally, because the natural history is not well characterized it is difficult to identify meaningful clinical outcome measures for potential therapeutic interventions.

A variety of clinician-entered patient registries for NF have been previously described [3,4]. Because the clinical manifestations of the neurofibromatoses are protean, and even pathological classification of some of the lesions is a subject of debate, it has been argued that only clinician-entered data could be reliable. However, previous efforts to validate the utility of patient-entered data in the neurofibromatoses [5] as well as the present analysis indicate that patient-entered data can be reliable for reporting symptoms of these disorders to the extent required for clinical trial eligibility screening.

In general, disease registries have been used to enable clinical trial recruitment, quality of life studies, outcome studies, post-market surveillance, development of disease management guidelines and siting of clinical trials [6]. Types of registries include population-based (such as cancer registries), disease-specific, national health database [7] or consortium-based such as the Rare Disease Clinical Research Network (RDCRN) Contact Registry [8], which contains 22 disease groupings representing over 200 rare diseases. Another model, disease agnostic, is exemplified by ResearchMatch [9], a project of the Clinical Translational Science Awards (CTSA) consortium, which allows any adult in the United States to enroll and potentially be matched with a clinical trial.

Patient registries may be organized under a governmental agency, a commercial enterprise such as pharmaceutical or biotech company, a private company specializing in patient recruitment, a hospital, clinic or physician, a non-profit or patient advocate group (PAG), or in partnership between one or more of these types of entities [7].

Numerous patient advocacy groups (PAGs) have established registries on their own initiative in order to further research in a specific condition. Such databases have the advantage of drawing on patient input in order to focus registry questions on what is important to them [10].

In 2012, CTF initiated recruitment to a web-based patient-entered registry (www.nfregistry.org) [11] to inform patients of research opportunities and to connect patients with investigators interested in recruitment to approved trials, regardless of institutional affiliation. Utilization of the registry for recruitment to observational, survey and interventional trials began in 2013.

In the present analyses, we compare features of patients in this self-reported registry with those of other patient- and clinician-reported NF registries and document its use as a resource for investigators.

## Methods

### Organization and recruitment to the registry

The registry questionnaires [S1 Text] and consent language [S2 Text] were developed in collaboration with two clinicians who are co-authors of this paper (BK, DV). The questionnaires were piloted in 50 patients and minor modifications were made. The NF registry protocol and informed consent were approved by the Western Institutional Review Board on April 16, 2012. It is Study Number 1131422, WIRB Protocol Number 20120455. All WIRB-approved research is required to be conducted research in accordance with the protocol, applicable laws and regulations, and the principles of research ethics as set forth in the Belmont Report. All participants or their parent/legal guardians provided online informed consent prior to entering any data in the database.

Recruitment took place through the CTF website, NF-related Facebook groups, at the annual CTF Patient Forum, through NF clinics and the CTF volunteer network, and through word of mouth and social media.

All registrants were required to supply name, sex, date of birth, race/ethnicity, mailing address, phone number and email address. The purpose of collecting race and ethnicity data was to fulfill Western Institutional Review Board reporting requirements, and to identify gaps in recruitment of specific groups. All registrants completed a demographics and family history section. Depending on the type of NF reported, they were directed to a specific questionnaire for NF1, NF2 or SCHW. Disease-specific questionnaires captured features of current published diagnostic criteria [12,13,14] and a variety of disease manifestations. They also posed quality of life questions. Participants were offered options with regard to being contacted by registry coordinators, including no contact.

For purposes of the present analyses, studies that have recruited through the Registry are classified as either observational; survey (i.e., patient-reported outcomes or quality of life); or interventional. Interventional studies are divided into psycho-social interventions and drug/device interventions. Data are available regarding how many registrants opened e-mails inviting them to participate in a study; however, data regarding the proportion that contacted the investigator, were screened or actually enrolled are not available.

#### Information collected

The criteria for diagnosis of NF1 were published by an NIH consensus development conference in 1988 [12]. The criteria for diagnosis of NF2 are the modified NIH consensus criteria as described by Evans in 2009 [13]. The criteria for diagnosis of SCHW are those detailed by Plotkin et al. in 2011 [14]. These criteria were used to create curation guidelines used by the Registry host (once known as PatientCrossroads, rebranded as AltaVoice, and recently acquired by and known as Invitae [15] to assign categories of “Criteria Met” or “Criteria Not Met” to each participant account. Participants were invited to upload copies of their genetic testing results to their profiles. In addition to soliciting information related to confirmation of their diagnoses, the presence and age at onset of other probably or possibly NF-related symptoms were collected. Registrants were also asked to identify their key concerns related to their disorder.

### Registry platform

#### Data storage, security and confidentiality

A state-of-the-art web-based, patient opt-in registry (maintained by Invitae) [15] was used for data capture. Patients or parent/guardians of minor patients provided answers to a disease-specific questionnaire via a secure web portal. The registry website could be accessed on mobile devices but was not optimized for them. Although the current questionnaires are currently available only in English, translation of the registry into multiple languages is planned.

HIPAA privacy security rules are enforced by policies and procedures that protect the confidentiality and security of protected health information. The database design is such that participant identifying information is stored in data tables separate from participant medical information.

Access to patient identifying information is restricted to authorized personnel at CTF and Invitae. Additional database security features of this registry target multiple levels, including data element (e.g., restricted access to fields), user (e.g., password authentication access), application (e.g., role-based access to features, access audit trails) and hosting services (e.g., firewall, secure sockets layer).

Invitae uses MySQL Server as the back-end relational database. All server requests are transmitted over SSL and use several layers of data and access protection, with a dedicated, managed Cisco router firewall and a redundant array of independent disks to ensure data recovery if a hard drive fails between backups. Back up (system-level and database-level) performed nightly is retained for 30 days with monthly backups stored for 1 year at the data center.

#### Approval and execution of requests for use

Information on applying for access to de-identified data or for trial recruitment is available on the NF Registry website [16]. Requests for registry use are reviewed by the CTF Clinical Program Director and by the Data Access Committee, a group comprised of NF experts and NF patients and family members. All studies and recruitments must be approved by the investigator’s Institutional Review Board. Only de-identified data can be shared by CTF with investigators.

Following approval by the Data Access Committee, The CTF Clinical Program Director extracts from the database a list of registrants reporting the manifestation(s) of NF specific to the study and any other specified characteristics such as age range or geographic location. Search results are used to generate an email list of potentially eligible participants, who receive an email from sent from the Invitae platform. The text provides a lay-level description of the study and the investigator’s contact information. The email advises the recipient to contact his or her healthcare provider to ask whether the study may be suitable for them. The registrant may initiate contact with the investigator to explore study participation.

#### Curation and quality control

Data curation was carried out by Invitae according to guidelines set by CTF based on current diagnostic criteria [12,13,14]. Each registrant was assigned a status of “Criteria Met” or “Criteria Not Met” according to disease-specific criteria. Participants with NF2 and SCHW were additionally asked “Have you been diagnosed with [disorder] by a doctor” or, in the case of NF2, whether an *NF2* mutation had been identified by molecular testing. (In the case of NF1, genetic testing was not required for “Criteria Met” status because NF1 is most often diagnosed by clinical signs.) Curation also entailed querying registrants by email or telephone for incomplete or incorrect data such as implausible or impossible birthdates. Periodically, all registrants were sent email reminders to update their information.

### Methods for statistical analysis

A dataset for the present analysis was frozen as of October, 9, 2015 by extracting data from all accounts assigned a post-curation status of “Criteria Met” to Excel spreadsheets [S3 Table] using the reporting modules built into the registry. The spreadsheets were converted to text files (comma-separated) and imported into the R Statistical Environment V 3.2.4 [17]. Tabulations were performed using the stat.table function of the Epi package [18]. Statistical analyses were performed using the Kruskal-Wallis and Wilcoxon signed rank tests (for continuous variables) and ordered categorical variables and the Chi^-^Square Test (for unordered categorical variables). In comparing more than 2 categories, the approach was to first perform an omnibus test of the entire set; if that test was significant then the categories were tested pairwise against each other. A *p* value of < 0.05 was considered statistically significant and *p* values were not adjusted for multiple comparisons.

Unless otherwise specified, when participants entered or updated their data in multiple sessions over time, the data used for analysis was the most recent data entered.

## Results

### Results across disorders

To validate or de-validate the hypothesis that the registry data would be valuable, a rigorous statistical analysis was carried out on a subset of 4680 participants who met data curation criteria based on current practice [12,13,14] for their disorder. This dataset contained records from the inception of the registry on June 10, 2012, to October 9, 2015. The 4680 participants had completed 6504 survey sessions. A single session was obtained for 3401 individuals and 1279 completed more than one session. In 50% of cases the registration was a self-report and in 47.4% of cases the report was made by a parent or guardian.

Participants who updated their surveys generally did so in response to a yearly reminder email, based on the observation that, overall, 74% of 1279 sessions subsequent to the first were made within 30 days of receiving a reminder. The strength of this association decreased with increasing numbers of sessions. Thus, 79% of second sessions, 69% of third sessions, 61% of 4th sessions and 36% of 5th sessions were made within 30 days of a reminder. Of those with one or more updating sessions, the median follow-up time (time between updates) was 402.35 days.

#### Sex

Table 1 shows enrollment by sex and type of NF. NF1 patients represented 86%, NF2 represented 12% and SCHW represented 2%. For each type, females were a majority of the registrants (56.2, 59.8, and 59.3% respectively).

**Table 1:** Enrollment by type of NF and sex.

Type	Female (Sex %)	Male (Sex %)	Total (Disorder %)
NF1	2261 (56.2)	1759 (43.8)	4020 (85.8)
NF2	346 (59.8)	233 (40.2)	579 (12.3)
SCHW	48 (59.3)	33 (40.7)	81 (1.9)
Total	2655 (56.7)	2025 (43.3)	4680 (100)

#### Age

The age of registrants at the time of registration ranged from < 1 year to 77 years. Among registrants with NF1 or NF2, females were significantly older than males (for NF1 median age 26.3 and 20.0 years respectively, Wilcoxon test *p* < 0.001, for NF2 median age 35.9 versus 30.0 years, *p* < 0.001). Among those with SCHW, the female and male proportions (median ages 43.6 and 46.7 years) did not differ significantly.

Registrants were asked to designate, if known, their age of diagnosis in one of 5 age groups. Their responses are shown in Table 2. 66.5% of registrants with NF1 were diagnosed before the age of 5, while 76.5% with NF2 were diagnosed after the age of 10 and 90.1% those with SCHW after the age of 20. Differences among the median age at diagnosis were highly significant (*p* < 0.001 for Kruskal-Wallis test among all 3 groups and Wilcoxon rank-sum test for subsequent pairwise comparisons among all pairs of disorders).

**Table 2:** Enrollment by age at diagnosis and type of NF.

Age Group (Years)	NF1 (%)	NF2 (%)	SCHW (%)	Total (%)
Less than 5	2635 (65.5)	54 (9.3)	0	2689 (57.5)
5–9	482 (12.0)	74 (12.8)	1 (1.2)	557 (11.9)
10–20	432 (10.7)	195 (33.7)	6 (7.4)	633 (13.5)
Over 20	348 (8.7)	248 (42.8)	73 (90.1)	669 (14.3)
Uncertain	123 (3.1)	8 (1.4)	1 (1.2)	132 (2.8)
Total	4020 (100)	579 (100)	81 (100)	4680 (100)

The cohort as a whole is biased toward pediatric patients. This is especially true for NF1, with 48% of NF 1 registrants under age 18. The other 2 disorders are less biased toward pediatric cases in the Registry, with 16% of NF2 patients and 1% of SCHW under 18.

#### Family history

At least 1 affected family member was reported by 41.9% of NF1 registrants, but only 25.2% of NF2 and 23.5% of SCHW registrants. Overall, 39.6% of the registrants had at least one other affected family member. 47.5% of registrants denied knowing of any other affected family member. The remainder was uncertain of their family history.

The majority (77.9%) of registrants was white, 5% were African-American and 4.1% were Asian. (Table 3).

**Table 3:** Enrollment by type of NF and race.

Race	NF1 (%)	NF2 (%)	SCHW (%)	Total (%)
White	3116 (85.2)	465 (12.7)	67 (1.8)	3648 (77.9)
African-American	224 (95.7)	10 (4.3)	0 (0)	234 (5.0)
Asian	159 (82.8)	30 (15.6)	3 (1.6)	192 (4.1)
Native American or Inuit	115 (88.5)	10 (7.7)	5 (3.8)	130 (2.8)
Pacific Islander	43 (82.7)	8 (1.9)	1 (1.9)	52 (1.1)
Other	287 (87.0)	39 (11.8)	4 (1.2)	330 (7.1)
Unknown	76 (80.9)	17 (18.1)	1 (1.1)	94 (2.0)
Total	4020 (85.9)	579 (12.4)	81 (1.9)	4680 (100)

The large majority, 86.8% of registrants, was from North America. 6.5% were from Europe while 3.0% were from Australia, New Zealand or a Pacific island. The geographic distributions were similar for the three disorders (Table 4). Among the 306 Europeans in the registry 281 (91.8%) were from countries in the European Union.

**Table 4:** Enrollment by geographic area and type of NF.

Geographic Area	NF1 (%)	NF2 (%)	SCHW (%)	Total (%)
North America	3543 (88.1)	455 (78.6)	66 (91.8)	4064 (86.8)
Europe	234 (5.8)	60 (10.4)	12 (14.8)	306 (6.5)
Australia-Oceania	121 (3.0)	21 (3.6)	0	142 (3.0)
Asia	43 (1.1)	16 (2.8)	1 (1.2)	60 (1.3)
South America	58 (1.4)	22 (3.8)	2 (3.0)	82 (1.8)
Africa	12 (0.3)	2 (0.3)	0	14 (0.3)
Caribbean	9 (0.2)	3 (0.5)	0	12 (0.3)
Total	4020 (100)	579 (100)	81 (100)	4680 (100)

#### U.S. registrants in comparison with U.S. Census data

Of the 4680 registrants in this report, 3904 (83.4%) were from the United States. In Table 5, we compare selected characteristics with current estimates from the U.S. Census Bureau [19]. The proportion of female registrants was higher than that in the U.S. population for all disorders, and was statistically significantly higher for those with NF1 and NF2. A similar result was seen among U.S. whites. U.S. African-Americans were less common among registrants than in U.S. Census estimates and the difference was statistically significant for all 3 types of NF. Although few in numbers, Native Americans were more frequent in the registry than in the Census estimates and the differences were statistically significant for NF1 and for SCHW.

**Table 5:** Selected population characteristics of U.S. registrants compared to U.S. Census estimates.

	NF1 (N)	p*	NF2 (N)	p*	SCHW (N)	p*	U.S. Population (N)
Female	55.7% (1902)	< 0.001	61.0% (258)	< 0.001	54.7% (35)	0.64	50.8% (163 280 761)
White	79% (2698)	< 0.01	85.8% (363)	< 0.001	82.8 (53)	0.37	77.1% (247 813 910)
Black or African- American	6.3% (215)	< 0.001	2.4% (10)	< 0.001	0	0.002	13.3% (42 748 704)
Native American or Inuit	3.2% (108)	< 0.001	1.9%(8)	0.17	7.8% (5)	< 0.001	1.2% (3 857 026)
Total	100% (3417)		100% (423)		100% (64)		100% (321 418 820)

p* = Binomial test versus proportion in U.S. population

### Specific symptomatologies

#### Neurofibromatosis type 1

The most commonly reported manifestations of the 4020 registrants with NF1 (Table 6) were cutaneous neurofibromas (65.8% of registrants), learning disabilities (50.1%), Lisch nodules (45.3%), NF1-related pain (42.8%) and plexiform neurofibromas (34.5%). Of the 2645 with at least 1 cutaneous neurofibroma 956 (36.1%) had between 1 and 10, 920 (34.7%) between 11 and 100 and 769 (29.1%) more than 100.

**Table 6:** Selected past or current manifestations of NF1 among 4020 registrants.

Manifestation	Present (%)	Absent (%)	Unsure (%)
Cutaneous neurofibromas	2645 (65.8)	1209 (30.1)	166 (4.1)
Any learning disability	2048 (50.1)	1063 (26.4)	532 (13.2)
Lisch nodule	1823 (45.3)	1353 (33.6)	844 (20.1)
NF1-related pain	1719 (42.8)	2301 (57.2)	--
Plexiform neurofibromas	1390 (34.5)	1598 (39.8)	1032 (25.7)
Bone fracture	922 (22.9)	2935 (73.0)	163 (4.1)
Spinal or paraspinal neurofibromas	876 (21.7)	2501 (62.2)	643 (16.0)
Optic gliomas	763 (19.0)	2596 (64.6)	661(16.4)
Long bone bowing	492 (12.2)	2995 (74.5)	533 (13.3)
Attention Deficit Disorder	258 (6.4)	3230 (80.3)	532 (13.2)
Malignant Peripheral Nerve SheathTumors	78 (1.9)	3245 (80.7)	697 (17.3)

Some form of learning disability was identified by 60.3% of registrants and 6.4% of this group specifically identified that disability as Attention Deficit Disorder.

Bone fractures were not rare. Although of 4020 registrants with NF1, 2935 (76.1%) denied a history of fractures, at least 1 fracture was reported by 922 (23.9%) of them. 39 registrants indicated they had suffered more than 5 fractures and 163 reported they were unsure if they had had a bone fracture.

1052 of the female registrants identified themselves as being of childbearing age at the last date of contact with the registry, and 489 (46.5%) reported that they had been pregnant at least once.

A comparison of NF1 symptom incidences in the patient-entered NF Registry with those reported in the literature is shown in Table 7. The cited published studies all used cross-sectional data. One of the studies was longitudinal but used cross-sectional data.

**Table 7:** Frequencies of various manifestations of NF1 in clinician-reported studies compared with NF registry.

Data Source	YEAR	ADULTS/ CHILDREN	Self/ Clinician	Single/ Multicenter	SUBJECTS	PN %	OPG	MPNST	FRECK	LISCH	TIBIAL	LD/ ADHD
**CTF Registry**	**2015**	**A & C**	**S**	**M Intl**	**4020**	**35**	**19**	**2**		**45**	**7**	**51**
Friedman [4]	1997	A & C	C	M Intl	1728	23	NA	5	NA	59	2	
Huson [20]	1989	A & C	C	S Wales	135	32	1.5		70	85	4	33
McKeever [21]	2008	C	C	M N Ireland	75		6.7		42	6	5.3	49
Cnossen [22]	1998	C	C	S Rotterdam	150	26.6	11.3		85.3	52	2	
Nemethova [23]	2013	Collection of NF	C	S Slovakia	108		31		85	36		
Overweg-Plandsoen [24]	1997	C & A	C	S Netherlands	196		10		92	93		
Boulanger [25]	2005	C	C	S Montreal	279	24.7	14.7	1.8		59.6		

PN = Plexiform neurofibroma; OPG = Optic pathway glioma; MPNST = Malignant peripheral nerve sheath tumor; FRECK = skin-fold freckling; LISCH = Lisch nodules; LD/ADHD = Learning disability/Attention Deficit Hyperactivity Disorder.

#### Neurofibromatosis type 2

The most common manifestations identified by the 579 NF2 registrants (Table 8) included difficulties with balance (81.3%), vestibular schwannomas (79.6%), tinnitus (74.8%), hearing loss (73.4%) and NF2-related pain (67.5%).

**Table 8:** Selected manifestations of NF2 among 579 registrants.

Manifestation	Yes (%)	No (%)	Unsure (%)
Balance problems	471 (81.3)	108 (18.6)	--
Vestibular schwannomas	461 (79.6)	54 (9.3)	64 (11.1)
Tinnitus	433 (74.8)	146 (25.2)	--
Hearing loss	425 (73.4)	154 (26.6)	--
NF2-related pain	391 (67.5)	188 (32.5)	--
Facial weakness	329 (57.5)	250 (43.2)	--

There were also a variety of other reported tumors including 303 (52.3%) meningiomas, 97 (16.8%) ependymomas, 293 (50.6%) spinal schwannomas, 156 (26.9%) neurofibromas, 23 (4.0%) optic gliomas and 72 (12.4%) otherwise unspecified neural tumors.

Hearing loss of some degree was reported by 411 of the NF2 registrants, of whom 72 (17.5%) indicated mild, 67 (16.3%) moderate and 58 (14.1%) severe loss. 214 (52.1%) indicated that they were deaf.

Juvenile cataracts were reported by 93 (16.1%) of 402 NF2 registrants who responded to this question. Among this group, 64 (68.8%) reported unilateral cataracts. Visual loss of some degree was reported by 389 (67.2%) of NF2 registrants, of whom 154 (39.6%) indicated mild, 162 (41.9%) moderate and 61 (15.7%) severe loss. 12 indicated that they were blind.

329 (57.5%) of 579 registrants with NF2 reported at least mild facial weakness and 77 (13.3%) considered it severe. Women were more likely to complain of facial weakness (60.1% of 346 women versus 51.9% of 233 men), but the difference fell just short of statistical significance (Chi-Square = 3.47, *df* = 1, *p* = 0.06).

When asked the nature of the NF2-related problem that the registrant considered major, hearing difficulty was by far the most commonly indicated (33% of the 579), followed by tumor burden (13.6%), dizziness (12.3%) and pain (11.9%).

#### Schwannomatosis

81 registrants indicated they had a diagnosis of SCHW and 72 of them indicated that a health care practitioner had confirmed the diagnosis. Of the 81 reporting a diagnosis of SCHW, 67 reported that a brain MRI was performed to check for the presence of vestibular schwannomas. 2 reported unilateral and 1 registrant reported bilateral vestibular schwannomas. For registrants with SCHW, multiple tumors, including meningiomas, ependymomas, and non-vestibular schwannomas, were the rule. Only 3 of the 81 indicated that they had only a single tumor.

11 reported that they had tested positive for a mutation in either *INI1*/*SMARCB1*/*SNF5* or *LZTR1*. 9 reported being tested without the finding of a known mutation in any of these genes.

71 (87.7%) of 81 SCHW registrants noted pain they ascribed to their disease. For 38 (47%) the pain was described as severe and for 13 (18.3%) as unmanageable.

### Research usage of the database

Between April 26, 2013 and July 8, 2016, 18 studies recruited subjects through the CTF Registry (Table 9). Of these, 12 were observational and 6 interventional. The observational studies involved focus groups for survey development and surveys regarding quality of life issues for patients and family members of patients with NF. The interventional studies included two Phase II drug trials and one radiotherapy trial. Some studies recruited subjects with all 3 of the neurofibromatoses and others sought subjects with specific disorders such as plexiform neurofibroma, MPNST, tibial bowing in NF1 or breast cancer in NF1. All interventional studies were performed in the US. One observational study was conducted in the UK.

**Table 9:** Studies using the registry for recruitment.

	Start Date	Population	Type of study	Email sent	Email opened(%)
1	Apr 2013	NF1, Tibial bowing	Observational	256	10541%
2	Mar 2014	NF1, NF2—Adult	Intervention: Behavioral	1465	5538%
3	May 2014	NF1, NF2—Adolescent	Observational: Focus Group	813	24730%
4	Sep 2015	NF1, NF2—Adolescent	Intervention: Behavioral	1840	59532%
5	Jul 2015	NF1, ages 16–34, plexiform neurofibroma	Intervention: Behavioral	1019	27127%
6	May 2015	NF1, ages 3–31, MPNST	Intervention: Drug -Phase II	668	29042%
7	Mar 2015	NF2, ages 12–40,vestibular schwannoma	Intervention: Drug –Phase II	141	4733%
8	Dec 2014	NF1, ages2-18,plexiform neurofibroma	Observational- Focus group	366	10128%
9	Apr 2015	NF1 ages 8–12, plexiform neurofibroma	Observational- Focus group	154	4429%
10	Jun 2015	NF1 ages 5–7,plexiform neurofibroma	Observational- Focus group	640	25139%
11	May 2013	NF1 ages 7–16	Observational	500	27254%
12	Mar 2014	NF1, MPNST*	Intervention: Radiation	39	1846%
13	Jul 2013	NF1, breast cancer	Observational	3	267%
14	Feb 2015	NF1, parents of affectedchildren	Observational	1605	65741%
15	Mar 2016	NF1, Adult, UK,plexiform neurofibroma	Observational: QoLquestionnaire development	37	12 32%
16	Mar 2015	NF1, pain	Observational: Questionnairedevelopment	3187	113335%
17	Oct 2015	NF1, NF2, SCHW	Analysis of registry data,clinic accessibility	4617	NA
18	Sep 2015	NF1 pediatric	Observational: QoL fieldtesting	3574	118833%

MPNST* = Malignant Peripheral Nerve Sheath Tumor.

The median proportion of potentially eligible subjects who opened the recruitment emails was 35.5% (mean 38.2, range 27–66%). In contrast, 2 bulk mailing services [26, 27] both report an average opening rate of 22% for health-related mass emailings. There were no clear differences in the proportions of subjects who opened emails regarding observational or interventional studies. Anecdotally, investigators have reported that they are pleased with the results of recruitment through the registry but there are no data regarding what proportion of registry subjects who opened emails actually contacted investigators, were screened or were enrolled in the studies.

### Clinical trial participation

Registrants were asked whether they had participated in a clinical trial for their condition. Of the cohort of 4680 in the main analysis (registered before October 9, 2015) 165 people with NF1 (4.1%) and 102 people with NF2 (18.8%) reported having been enrolled in a clinical trial. SCHW patients were asked if they wanted to be contacted by the International Schwannomatosis Database [28], a clinician-entered database, and 78 of these patients answered yes.

## Discussion

Disease registries have been used to support clinical research since at least the 1940s [29]. They may take the form of national health databases, contact registries [7], consortium-based, such as the NIH Office of Rare Diseases Research’s Rare Disease Clinical Research Network (RDCRN) Contact Registry [8], or disease agnostic, such as the Clinical Translational Science Awards (CTSA) consortium’s ResearchMatch [9].

In addition to their functions in clinical trial recruitment, quality of life studies, outcome studies, post-market surveillance, development of disease management guidelines and recruitment for and siting of clinical trials [6] they may be used to explore disease incidence, comorbidities and mortality, or for data-mining and hypothesis generation [29].

As a patient advocate organization, CTF’s aim in creating the NF Registry was to facilitate treatment and treatment-enabling research in this neglected disease area by developing a pool of recruitable patients. We saw a need for broad geographic reach to accumulate subgroups of patients with specific manifestations that could be targeted for clinical trials for NF, and to give all stakeholders (industry, academia, government) the ability to assess the distribution of the different manifestations of all forms of NF. Maintaining a patient-entered contact registry was seen by the Foundation as a way to promote treatment development in NF by de-risking the critical task of completing trial enrollment, which is especially difficult in rare disease [7]. In electing to create a patient-entered contact registry for NF, the Foundation’s aim was to strike a balance between the costly and time-intensive approach of compiling clinician-entered data and the urgent need to speed treatment development via a large and expanding database of patients unimpeded by geographic or institutional boundaries.

The CTF Registry has been successful in enrolling large numbers of NF1 subjects and smaller numbers with NF2 or SCHW. Most participants reside in the US. Recruitment through US-based NF clinics and meetings may account for this predominance. When compared to U.S. Census data [19], registrants were more likely to be female and white than the population as a whole. The fact that the website was available in English only may have reduced enrollment of non-English speakers both in the U.S. and abroad.

The dominance of pediatric patients in the cohort is likely due to the fact that NF1 is usually diagnosed at young ages due to pathognomic features that begin to appear at or shortly after birth while NF2 and SCHW are generally diagnosed in adolescents and adults. Nonetheless, the need for more adult registrants will be addressed in future recruitment efforts.

The CTF Registry is currently the largest reported NF1 cohort in the literature and the only one that was designed to be available generally to interested investigators. In general, the manifestations reported by CTF registrants with NF1 occurred at rates within the range reported by other sources. However, virtually all registries have some biases based on the nature of their respective recruitments. Registries that are limited to children are likely to have fewer registrants with the types of manifestations that become more prevalent with age, and registries that are hospital- or clinic-based may be biased toward subjects with more severe manifestations of the disorder.

For cutaneous neurofibromas, the overall prevalence reported in the registry was only 65.8%; however, stratifying respondents by age showed a more expected higher prevalence. By age 30, 81.8% reported cutaneous neurofibromas, by age 40, 95.7%, and by age 50, 98.2. NF2 is often not diagnosed until adulthood, which is consistent with our finding that 76% of registrants were diagnosed after age 10. Evans et al. [30] reported the presenting symptoms in 120 patients with NF2. They found unilateral or bilateral hearing loss in 44% and balance dysfunction in 8%. The rates of these problems may have been higher in the CTF registrants who were older and had developed more disease manifestations over time.

We are aware of the limitations of the current data set. Namely, patients may not know, may mistake, or may fail to recall the details of some of the information asked in the surveys. However, when we compared the frequencies of the various manifestations of NF1 in clinician-reported studies to the frequencies extracted from the NF Registry, we observed that in most instances the frequencies match very closely [Table 7]. We therefore feel confident that the registry data is of sufficient quality to be used for clinical trial recruitment.

Moreover, it is the responsibility of the investigator of the individual trial to confirm that the patient meets entry requirements. We do have the ability to re-contact and re-consent participants to develop further this cohort in the future, and are open to expanding registry capabilities uses to include, for example, outcomes studies and natural history studies.

The Foundation is, however, committed to ongoing monitoring of the registry entries to continuously improve the quality of our patient-entered data. Following the present analysis, we improved the disease surveys to clarify items that appeared to be prone to misunderstanding. We added a glossary and photographs of some of the major manifestations. In addition, the Foundation is investing resources in patient engagement to ensure that NF patients are fully educated about their manifestations. In light of the positive effect of reminder emails shown by the proximity of updates to reminder emails (see Results), we are increasing the frequency of these emails to at least every 6 months instead of yearly.

A particularity of NF is that as a lifelong condition that may not be at all obvious to casual observers, and some patients and affected families may wish to keep their NF status confidential for fear of stigma, employment discrimination, or insurance limitations affecting their well-being. Anecdotally we have found some resistance to online disease registrations due to concerns over internet privacy. As a patient advocacy organization, CTF works to overcome this reluctance by explaining privacy and security measures in place, building a sense of community and making the case for patient partnership in research. We present the registry at scientific and lay-oriented events; use CTF’s volunteer network to campaign on a local level about the importance of registering and updating; promote via NF-related Facebook pages and CTF newsletters; explore IRB-approved small financial incentives; and create patient engagement programs.

Thus far, the database has been useful to investigators in recruitment for observational trials, particularly for development of patient-reported outcomes for future use in clinical trials. There have been limited interventional trials available, particularly for NF1. Recruitment to trials may be geographically limited, making them unavailable to many registrants. This is reflected in the very small numbers of NF1 registrants who had participated in trials in this analysis.

It is anticipated that the number of clinical trials for these disorders will grow and the NF Registry will be of increasing value as both the number of registrants and the number of trials expand.

## Conclusions

The CTF-sponsored NF Registry is the largest reported NF database to date and continues to grow. As of December 27, 2016, it contains 7371 registrants from 71 countries. It is expected to continue to grow in number of participants and to continue to be supported by CTF funding and personnel for at least 5 years and likely longer. Though it does not contain clinician-entered data, it has the advantages of worldwide reach and access to patients who otherwise might be missed because they do not see a specialist for NF care. These factors make it valuable as a contact registry.

The aim of the statistical analyses of the dataset was validating or de-validating the hypothesis that patient-entered data about symptoms approximates the patterns seen in clinician-entered data, and that the NF Registry can therefore be dependably used for initiating patient recruitment for clinical trials.

Thus far, the database has been useful to investigators for recruitment of mainly observational trials, particularly those for development of patient-reported outcomes for future use in clinical trials.

From the analyses, we postulate that although self-reported, the incidences of NF manifestations in registrants are broadly comparable to what has been reported in clinician-reported databases. To date, the database has supported geographically diverse recruitment for 14 observational and 4 interventional trials. Examining the association between time of update reminder email with time of updating suggests that participants should be contacted more frequently with requests to update their information. Translation of the questionnaires to additional languages and active recruitment of underrepresented populations may make the registry data more representative of the U.S. NF population, broaden its utility internationally and increase the usefulness of the database to investigators. Patients are clamoring for more interventional studies and availability of additional studies will likely aid in expanding registration.

## Supporting information

S1 Text NF Registry questionnaires. (DOCX)

S2 Text Online informed consent. (DOCX)

S1 Table Dataset from Registry NF1, NF2, SCHW. Subset exported on October 9, 2015. NA = Not Available (blank or missing data). Where Patient ID (column B) is repeated, same participant answered questionnaire at >1 time point.
(XLSX)

S2 Table Definitions of headings in S1 Table. (DOCX)
