# Supplementary material for: Characterization and utilization of an international neurofibromatosis web-based, patient–entered registry: An observational study
Source: PLoS One. 2017 Jun 23;12(6):e0178639. doi: 10.1371/journal.pone.0178639 (PMC5482445; doi:10.1371/journal.pone.0178639)
Supplement: S1 Text — (DOCX) [file pone.0178639.s001.docx]

**Survey 1 - NF1**

**Family History**

**Has anyone in your family, living or deceased had NF1 beside you?**

| Yes No one else in my family has it I am not sure if anyone in my family has NF1 |
| --- |

**Family Member**

**Please select the number of family members that have/had NF1 that are living or deceased.**
**For example: If the mother of a child with the disorder has NF1, then 'Mom' would be selected.**

| **Answers** Dad Mom Son Daughter Brother Sister Half-sister (mom's side) Half-brother (mom’s side) Grandmother (mom’s side) Grandfather (mom’s side) Aunt (mom’s side) Uncle (mom’s side) Cousin (mom’s side) Half-sister (dad’s side) Half-brother (dad’s side) Grandmother (dad’s side) Grandfather (dad’s side) Aunt (dad’s side) Uncle (dad’s side) Cousin (dad’s side) Grandson Granddaughter | **Number of family members** 0 1 2 3 4 5 6 7 8 9 10 + |
| --- | --- |

**Diagnosis**

**At what age was the diagnosis of NF1 made?**

| Under 5 years old 5 - 9 years old 10 - 20 years old Older than 20 years old Not sure |
| --- |

**Do you have cafe au lait spots (brown markings on the skin) and if so how many?**

| No Yes - fewer than 6 Yes - more than 6, less than 20 Yes - more than 20 Not sure if I have them |
| --- |

**Do you have freckles in your armpits?**

| No Yes - on one armpit Yes - in both armpits Not sure if I have them |
| --- |

**Do you have freckles in your groin?**

| No Yes - on one side Yes - on both sides Not sure if I have them |
| --- |

**Do you have Lisch nodules (pigmented markings in the iris of the eye) as confirmed by an eye doctor?**

| No Yes - in one eye Yes - in both eyes Not sure if I have Lisch nodules Yes, I have Lisch nodules but don't know in which eye |
| --- |

**Did you ever have a genetic test to confirm your diagnosis of NF1?**

| No Yes Not sure |
| --- |

**NF1 Gene**

**Has a mutation in your NF1 gene been identified?**

| I have a known NF1 mutation The mutation was not identified |
| --- |

**Tumor Type - Neurofibroma**

**Do you have neurofibromas (small bumpy tumors) on your skin and if so, how many?**

| No Yes - more than 1, less than 10 Yes - more than 10, less than 100 Yes - more than 100 Not sure if I have them |
| --- |

**Neurofibroma - Intervention/Therapy**

**How was the neurofibroma treated? (check all treatments you have had for these tumors)**

| Drug therapy Radiation therapy Surgery No treatment I am not sure |
| --- |

**Tumor Type - Neurofibromas Near Spine**

**Have you been diagnosed with neurofibromas adjacent to your spine as identified by MRI?**

| No Yes I am not sure |
| --- |

**Neurofibromas Near Spine - Intervention/Therapy**

**How was the neurofibroma adjacent to your spine treated? (check all treatments you have had for these tumors)**

| Drug therapy Radiation therapy Surgery No treatment I am not sure |
| --- |

**Tumor Type - Plexiform Neurofibroma**

**Have you been diagnosed with plexiform neurofibroma(s) and if so how many?**

| No Yes - just one Yes - more than one, less than 5 Yes - more than 5 Not sure if I have a plexiform neurofibroma |
| --- |

**Plexiform Neurofibroma - Location/Intervention/Therapy**

**Please specify location(s) of plexiform neurofibroma(s):**

| Head area Neck area Chest area Arms Legs Abdomen area I am not sure Spine Back |
| --- |

**How was the plexiform neurofibroma treated? (check all treatments you have had for these tumors)**

| Drug therapy (any kind of drug therapy:clinical trial or approved) Radiation therapy Surgery No treatment I am not sure |
| --- |

**Tumor Type - Optic Nerve Pathway Glioma**

**Have you ever been diagnosed with an optic nerve pathway glioma?**

| No Yes - on one side Yes - on both sides I am not sure if I have ever had an optic nerve pathway glioma |
| --- |

**Optic Nerve Pathway Glioma - Intervention/Therapy**

**How was the optic nerve pathway treated? (check all treatments you have had for these tumors)**

| Drug therapy Radiation therapy Surgery No treatment I am not sure |
| --- |

**Tumor Type - Malignant Peripheral Nerve Sheath Tumor (MPNST)**

**Have you ever been diagnosed with a malignant peripheral nerve sheath tumor (MPNST)?**

| No Yes I am not sure if I have ever had an MPNST |
| --- |

**Malignant Peripheral Nerve Sheath Tumor (MPNST) - Intervention/Therapy**

**How was the MPNST treated? (check all treatments you have had for these tumors)**

| Drug therapy Radiation therapy Surgery No treatment I am not sure |
| --- |

**Clinical Trial**

**Have you ever participated in a clinical trial for treatment of one of your NF tumors?**

| No Yes |
| --- |

**Please describe tumor.**

|  |
| --- |

**Please describe drug trial.**

|  |
| --- |

**Bone Abnormalities**

**Do you have or have you ever had bone fractures?**

| No Yes - 1 Yes - more than 1, but less than 5 Yes more than 5 Not sure |
| --- |

**Do you have or have you ever had osteoporosis?**

| No Yes Not sure |
| --- |

**Do you, or have you had sphenoid wing dysplasia (bone deficiency of the eye socket)?**

| No Yes - on one side Yes - on both sides I am not sure if I have had sphenoid wing dysplasia |
| --- |

**Do you have, or have you had, scoliosis (curvature of the spine)?**

| No Yes - upper spine Yes - thorax Yes - lower spine Yes - not sure where in the spine I am not sure if I have had scoliosis Yes - I have scoliosis in multiple areas of my spine |
| --- |

**Scoliosis Treatment**

**How was the scoliosis treated? (check all treatments you have had for this)**

| Bracing Fixation with rods Drug therapy No treatment I am not sure how it was treated |
| --- |

**Bone Bowing**

**Do you, or have you had, bowing of the long bones of the lower leg or forearm?**

| No Yes - one leg Yes - both legs Yes - one arm Yes - both arms I am not sure if I have had bowing of the long bones Yes - I have bowing in multiple areas |
| --- |

**Bone Bowing Treatment**

**How was the bone bowing treated? (check all treatments you have had for this)**

| Amputation Casting Drug therapy Surgery No treatment I am not sure how it was treated Brace |
| --- |

**Learning Difficulties**

**Do you have, or have you had, learning difficulties in school?**

| Yes - Learning disabilities Yes - Attention Deficit Disorder (ADD) No Unsure Yes - I have both ADD and Learning Disabilities |
| --- |

**Learning Difficulties Treatment**

**Have you had any of the following treatments/interventions for these? (check all that apply)**

| Extra tutoring in school or at home ADHD medication Participation in Lovastatin trial No treatment I am not sure if it was treated |
| --- |

**Itching**

**Have you ever suffered from itching?**

| Yes No Unsure |
| --- |

**Itching Treatment**

**If you have suffered from itching, did you receive treatment?**

| No Yes - but not sure which treatment |
| --- |

**If you had treatment, please rate how effective it is/was.**

| Very effective More or less effective Very minor effect Not at all effective Unsure Not applicable - did not receive treatment |
| --- |

**Other Clinical Manifestations**

**Do you have or have you ever had any of the following health concerns? (check all that apply)**

| High blood pressure Brain issues, such as stroke Heart problems Kidney problems Glomus Tumors Headaches (migraine) Hydrocephalus Cancer Seizures Early onset of puberty Late onset of puberty Poor weight gain in childhood Hormonal imbalance issues that affected your NF pregnancy or while taking contraception No, none of the above I am not sure if I have any of the above health concerns |
| --- |

**Do you have pain associated with your NF1 and if so have you been prescribed pain medication?**

| No - I do not get NF1 related pain Yes - I have NF1 related pain but take no prescribed medication Yes - I have NF1 related pain and I do take prescribed medication to treat it |
| --- |

**The following page lists questions specifically for females with NF1 and of childbearing age. Please confirm below.**

| Yes, I am a female of child bearing age and have NF1 Yes, I am a female but not of child bearing age No, I am not a female |
| --- |

**For Women Only**

**Have you ever had a diagnosis of breast cancer?**

| No - I have not had breast cancer Yes - I have had breast cancer in one breast Yes - I have had breast cancer in both breasts Not applicable |
| --- |

**Breast Cancer Treatment**

**How was your breast cancer treated? (check all treatments you have had)**

| Drug therapy Mastectomy Radiotherapy No treatment yet Not applicable Lumpectomy |
| --- |

**Pregnant**

**Have you ever been pregnant?**

| Yes No |
| --- |

**Pregnancy**

**If you have been pregnant, during the pregnancy**

| The number of neuromas increased The number of neuromas did not increase The neuromas grew in size The size of the neuromas remained the same No changes in neuromas were seen |
| --- |

**If you have been pregnant, during the pregnancy**

| Pain became more severe Pain was so severe that there was need for medication and/or surgery No changes in pain |
| --- |

**If you have been pregnant, did you have obstetrical complications?**

| No |
| --- |

**Other Health Related Problems**

**Are there are any other health related problems that weren't addressed in the survey that you would like to describe?**

**Survey 2- NF2**

**Family History**

**Has anyone in your family, living or deceased had NF2 beside you?**

| Yes No one else in my family has it I am not sure if anyone in my family has NF2 |
| --- |

**Family Member**

**Please select the number of family members, living or deceased that have/had NF2.**
**For example: If the mother of a child with the disorder has NF2, then 'Mom' would be selected.**

| **Answers** Dad Mom Son Daughter Brother Sister Half-sister (mom's side) Half-brother (mom’s side) Grandmother (mom’s side) Grandfather (mom’s side) Aunt (mom’s side) Uncle (mom’s side) Cousin (mom’s side) Half-sister (dad’s side) Half-brother (dad’s side) Grandmother (dad’s side) Grandfather (dad’s side) Aunt (dad’s side) Uncle (dad’s side) Cousin (dad’s side) Grandson Granddaughter | **Number of family members** 0 1 2 3 4 5 6 7 8 9 10 + |
| --- | --- |

**Diagnosis**

**Have you been diagnosed with NF2 by a doctor or healthcare provider?**

| Yes, I was diagnosed by a doctor Yes, I was diagnosed by another healthcare provider No, I have not been diagnosed by a doctor or healthcare provider |
| --- |

**At what age were you diagnosed with NF2?**

| Under 5 years old 5 - 10 years old 10 - 20 years old Older than 20 years old Not sure |
| --- |

**What was the first symptom associated with your NF2? (select only one)**

| Hearing loss Tinnitus (ringing/buzzing in the ear) Dizziness or imbalance Facial weakness Vision change Pain I am not sure |
| --- |

**NF2 Signs and Symptoms**

**Do you have two or more individual tumors?**

| Yes - I have two or more individual tumors No - I have only one individual tumor No - I do not have any tumors I don't know |
| --- |

**Do you have a vestibular schwannoma (i.e. one or more vestibular tumors on the side of the brain)?**

| No Yes, I have bilateral vestibular schwannomas (vestibular tumors on both sides of the brain) Don't know/not sure Yes, I have a unilateral vestibular schwannoma (a vestibular tumor on only one side of the brain) |
| --- |

**Other than vestibular schwannoma, have you had any other tumors? If so, which ones? (check all that apply)**

| Meningioma Ependymoma Spinal schwannoma Other schwannoma I am not sure Neurofibroma Glioma |
| --- |

**Do you have a juvenile cataract?**
Juvenile Cataract - Clouding of the eye in a young person

| Yes - I have a cataract in one eye Yes - I have a cataract in both eyes No - I do not have cataract I don't know |
| --- |

**Quality of Life**

**How would you rate your level of hearing loss in your LEFT ear? (Select only one)**

| None Mild Moderate Severe Deaf |
| --- |

**How would you rate your level of hearing loss in your RIGHT ear? (Select only one)**

| None Mild Moderate Severe Deaf |
| --- |

**How would you rate your level of dizziness/imbalance? (Select only one)**

| None Mild Moderate Severe |
| --- |

**How would you rate your level of tinnitus (ringing/buzzing in the ear)? (Select only one)**

| None Mild Moderate Severe |
| --- |

**How would you rate your level of facial weakness? (Select only one)**

| None Mild Moderate Severe |
| --- |

**How would you rate your level of pain associated with your NF2? (Select only one)**

| None Mild Moderate Severe |
| --- |

**How would you rate your level of vision change/loss associated with your NF2? (Select only one)**

| None Mild Moderate Severe Blind |
| --- |

**Which of the following would you rate as your biggest problem from NF2? (Select only one)**

| Hearing difficulty Dizziness/imbalance Facial weakness Tinnitus (ringing/buzzing in the ear) Problems with vision Pain Depression Tumor burden |
| --- |

**Intervention and Therapy**

**How many surgeries have you had to treat a vestibular schwannoma associated with your NF2?**

| None One Two Three Four Five More than five I am not sure |
| --- |

**How many surgeries have you had to treat a meningioma associated with your NF2?**

| None One Two Three Four Five More than five I am not sure |
| --- |

**How many surgeries have you had to treat a peripheral schwannoma associated with your NF2?**

| None One Two Three Four Five More than five I am not sure |
| --- |

**How many times have you received a treatment course of radiation/radiosurgery for an intracranial tumor (including vestibular schwannoma or meningioma) associated with your NF2?**

| None One Two Three Four Five More than five I am not sure |
| --- |

**Do you have any tumors which you and your doctors have chosen to observe (watch and wait) rather than treat with surgery, radiation, or chemotherapy? (check all that apply)**

| None Meningioma Ependymoma Spinal schwannoma Other schwannoma Spinal meningioma I am not sure |
| --- |

**Have you been genetically tested and if so has a mutation in your NF2 gene been identified?**

| I have never been genetically tested I have been tested and I have a known NF2 mutation I have been tested but the mutation was not identified I am not sure if I have been genetically tested |
| --- |

**Have you been implanted with a cochlear implant (CI)?**

| No Yes |
| --- |

**Cochlear Implant (CI)**

**How does the cochlear implant help you.**

| It does not help me at all It helps me somewhat There is a fair improvement It helps me very much It is extremely helpful to me |
| --- |

**Auditory brainstem implant (ABI)**

**Have you been implanted with an auditory brainstem implant (ABI)?**

| No Yes |
| --- |

**Auditory brainstem implant (ABI) - Part 2**

**How does the auditory brainstem implant help you?**

| It does not help me at all It helps me somewhat There is a fair improvement It helps me very much It is extremely helpful to me |
| --- |

**Trial Medication**

**Have you taken a trial medication specifically to treat tumors associated with your NF2?**

| No Yes |
| --- |

**Trial Medication Treatment**

**Which trial medications have you taken? (check all that apply)**

| Lapatinib Gefitinib (Iressa) Erlotinib (Tarceva) Bevacizumab (Avastin) Imatinib (Gleevec) PTC299 SOM230B |
| --- |

**Over-the-Counter Supplements**

**Have you taken over-the-counter supplements specifically to treat tumors associated with your NF2?**

| No Yes |
| --- |

**Over-the-Counter Supplements - Treatment**

**Which over-the-counter supplements have you taken? (check all that apply)**

| Caffeic Acid Phenethyl Ester (CAPE) Extracts (as found in Bio 30) N-acetylcysteine (NAC) Glutathione Whey protein Artepillin C (ARC) Bioperine Black Seed Oil Coenzyme Q10 (CoQ10) Curcumin from Turmeric Epigallocatechingallate (EGCG) from Green Tea Isothiocyanate Sulforaphane found in Broccoli 5-Loxin from Boswellia Omega-3 Resveratrol Vitamin C Vitamin D Vitamin E |
| --- |

**Other Health Related Problems**

**Are there are any other health related problems that weren't addressed in the survey that you would like to describe?**

|  |
| --- |

**Survey 3 - Schwannomatosis**

**Schwannomatosis Family History**

**Has anyone in your family, living or deceased, been diagnosed with schwannomatosis besides you?**

| Yes No one else in my family has it Not sure/family history unknown |
| --- |

**Schwannomatosis Family Member**

**Please select the family members, living or deceased, that have/had schwannomatosis.**
**For example: If the mother of a child with the disorder has schwannomatosis, then 'Mom' would be selected.**

| **Answers** Dad Mom Son Daughter Brother Sister Half-sister (mom's side) Half-brother (mom’s side) Grandmother (mom’s side) Grandfather (mom’s side) Aunt (mom’s side) Uncle (mom’s side) Cousin (mom’s side) Half-sister (dad’s side) Half-brother (dad’s side) Grandmother (dad’s side) Grandfather (dad’s side) Aunt (dad’s side) Uncle (dad’s side) Cousin (dad’s side) Grandson Granddaughter | **Number of family members** 0 1 2 3 4 5 6 7 8 9 + |
| --- | --- |

**NF2 Family History**

**Has anyone in your family, living or deceased, had NF2?**

| Yes, I have NF2 but no other family members have it Yes, I have NF2 and I have a family member that also has it No, I do not have NF2 and none of my family members have it No, I do not have NF2 but I am not sure if any other family have it |
| --- |

**NF2 Family Member**

**Please select the number of family members, living or deceased, that have/had NF2.**
**For example: If the mother of a child with the disorder has NF2, then 'Mom' would be selected.**

| **Answers** Dad Mom Son Daughter Brother Sister Half-sister (mom's side) Half-brother (mom’s side) Grandmother (mom’s side) Grandfather (mom’s side) Aunt (mom’s side) Uncle (mom’s side) Cousin (mom’s side) Half-sister (dad’s side) Half-brother (dad’s side) Grandmother (dad’s side) Grandfather (dad’s side) Aunt (dad’s side) Uncle (dad’s side) Cousin (dad’s side) Grandson Granddaughter | **Number of family members** 0 1 2 3 4 5 6 7 8 9 + |
| --- | --- |

**Diagnosis**

**Have you been diagnosed by a doctor that you have schwannomatosis?**

| No Yes Not sure |
| --- |

**At what age was the diagnosis of Schwannomatosis made?**

| Under 5 years old 5 - 9 years old 10 - 20 years old Older than 20 years old Not sure |
| --- |

**Have you had a genetic test that shows you have a mutation in the INI1/SmarcB1/Snf5 gene or in the LZTR1 gene?**

| No Yes, I was tested and I have the mutation Yes, I was tested and I do not have the mutation Don't know |
| --- |

**Have you had a genetic blood test that showed that you have an NF2 gene mutation?**

| No Yes, I was tested and I have the mutation Yes, I was tested and I do not have the mutation Don't know |
| --- |

**Do you have more than one schwannoma tumor?**

| No Don't know |
| --- |

**Have you had a brain MRI to check whether you have vestibular schwannomas/acoustic neuromas?**

| No Yes Don't know |
| --- |

**Vestibular Schwannoma/Acoustic Neuroma**

**Do you have a vestibular schwannoma/acoustic neuroma?**

| No Left ear Right ear Both ears Don't know |
| --- |

**Pain**

**Do you have pain?**

| No Yes |
| --- |

**Severity of Pain**

**How severe is the pain?**

| Mild Moderate Severe no medicine taken Severe - controllable with medicines Unmanageable |
| --- |

**Schwannomatosis International Database**

**The Schwannomatosis International Database (www.schwannomatosis.com) is a project in which doctors enter patient information in a database that can then be used for international research. Have you enrolled in this database through one of your doctors?**

| No Yes Don't know |
| --- |

**Research Participant**

**Would you be interested in being contacted by the Schwannomatosis International Database project about the possibly of participating in this initiative?**
If yes, contact Amanda Bergner, MS, CGC by email at abergne1@jhmi.edu or by phone 443-287-1914.

| No Yes |
| --- |

**Other Health Related Problems**

**Are there are any other health related problems that weren't addressed in the survey that you would like to describe?**

|  |
| --- |
