# Supplementary material for: Characterization and utilization of an international neurofibromatosis web-based, patient–entered registry: An observational study
Source: PLoS One. 2017 Jun 23;12(6):e0178639. doi: 10.1371/journal.pone.0178639 (PMC5482445; doi:10.1371/journal.pone.0178639)
Supplement: S2 Text — (DOCX) [file pone.0178639.s002.docx]

#9853357.3

Children’s Tumor Foundation

**Research Participant Information**

**Sheet Sponsor:** Children’s Tumor Foundation

**Protocol Title:** Neurofibromatosis (NF) Registry Funded by Children’s Tumor Foundation

**Investigator:** Pamela Knight, M.S., Clinical Program Manager, Children's Tumor Foundation

You are being asked to enter information into a patient registry because you or a child of whom you are parent or legal guardian has been diagnosed with neurofibromatosis (NF). The patient registry is sponsored by the Children’s Tumor Foundation, a non-profit organization dedicated to improving the health and well being of individuals and families affected by NF.

# What is the purpose of the NF Registry?

One purpose of this registry is to identify people with NF who may be interested in participation in clinical trials (research studies that evaluate experimental therapies) or other research studies being conducted in patients with NF. Another purpose of this registry is to determine how common specific characteristics of NF are.

# What will happen if I take part in this registry?

1. You will enter your name and contact information (and the name of the person for whom you are entering information, if you are doing so on behalf of a child), date and city of birth, race, ethnicity, gender, and the type of NF with which you have (or your child has) been diagnosed.
2. You will be asked to answer a series of specific questions about your NF. Answering these questions will take less than 30 minutes. You do not have to complete all survey questions at one time; any information you have entered will be saved, and you can complete any remaining questions when you log into the registry again. Once a year, you will be asked to update any information that has changed.
3. You will be asked if the Children’s Tumor Foundation may contact you
   1. If they have any additional questions regarding information you have entered.
   2. If there is a future opportunity to participate in a clinical trial or research study.
   3. If you would like further information about NF research news or events.

# What if I answer, “yes” to being contacted about clinical trials or research studies?

The Children’s Tumor Foundation will see if there are clinical trials or research studies that are looking for people in your age group and with NF characteristics like yours. If they find a match, The Children’s Tumor Foundation will provide you with contact information at the institution conducting the research, so you may get in touch with them directly.

Providing your contact information and entering information about your NF into the registry does not mean that you will qualify for participation in a clinical trial or research study. It will be up to the institution conducting the research to determine if you qualify for participation.

# How else will my information be used?

The information you provide about your NF will be summarized along with data from other registry participants so that the Foundation and others interested in research on NF can understand how common specific characteristics of NF are, and what treatments are being used. The Children’s Tumor Foundation may share your data with individuals or institutions conducting clinical trials or research studies, companies developing potential drugs or other treatments for NF, or other parties involved in research of patients with NF, but any information that identifies you will be removed. Your information may also be disclosed to Western Institutional Review Board® (WIRB®). This information is shared so the research can be conducted and properly monitored.

# Are there any risks to participating in the registry?

There is the potential risk of loss of confidential information. Keeping the data collected from your survey in a secure computer database will minimize this risk. Only people at the Children’s Tumor Foundation will have direct access to the information that is collected. Your name or other information that may identify you will not be included in any data shared with parties outside the Children’s Tumor Foundation. If a study using this information is published or presented at scientific meetings, your name or other information that might identify you will not be included.

# Are there benefits to participating in the registry?

You may not receive a direct benefit if you agree to participate. You may be informed about clinical trials that may offer benefit to you. In the future, NF patients may benefit from the information you have provided to the registry.

# What happens if I don’t participate?

Your participation in the registry is voluntary. You will not be penalized or lose benefits if you decide not to participate. The alternative to participating in the registry is not to participate.

# Who may I contact if I have questions about participating in the registry?

You may contact Pamela Knight, M.S. of the Children’s Tumor Foundation at 1-212-344- 6633, ext. 8555, for questions about the registry.

You may contact the Western Institutional Review Board (WIRB) at 1-800-562- 4789 if you have questions about your rights as a research subject. WIRB is a group of people who perform independent review of research.

For technical questions, please contact PatientCrossroads at [ntfregistry@patientcrossroads.org.](mailto:ntfregistry@patientcrossroads.org.)

# How long will my participation last?

This permission for contact and use of your registry data will not end unless you cancel it. You may cancel it by sending written notice to Pamela Knight, M.S., at the Children’s Tumor Foundation, at [pknight@ctf.org](mailto:pknight@ctf.org), or at the address below.

However, the data that has been uploaded prior to cancelation will remain in the database.

Pamela Knight, M.S.

Clinical Program Manager

Children's Tumor Foundation

95 Pine Street, 16th Floor

New York, NY 10005
