# Supplementary material for: Characterization and utilization of an international neurofibromatosis web-based, patient–entered registry: An observational study
Source: PLoS One. 2017 Jun 23;12(6):e0178639. doi: 10.1371/journal.pone.0178639 (PMC5482445; doi:10.1371/journal.pone.0178639)
Supplement: S2 Table — (DOCX) [file pone.0178639.s004.docx]

S2 Table.

**Definition of Headings in S1 Table**

**NF1**

| A | NF1RowNo | row number of patient |
| --- | --- | --- |
| B | PatientID | unique patient identifier |
| C | FamilyID | unique family identifier |
| D | SessionID | unique session identifier |
| E | ClinicalStatus | meets diagnostic criteria for NF1 |
| F | Relation to Case | patient or parent of patient |
| G | Syndrome | type of NF |
| H | Gender | male or female |
| I | CafeAuLait | have café au lait spots |
| J | ArmpitFreckles | have freckles in armpits |
| K | GroinFreckles | have freckles in groin |
| L | LischNodules | have Lisch nodules in eye |
| M | Neurofibromas | neurofibromas on skin, how many |
| N | NFRxDrugs | neurofibromas treated with drugs |
| O | NFRXRadiation | neurofibromas treated with radiation |
| P | NFRxSurgery | neurofibromas treated with surgery |
| Q | NFRxNone | no treatment of neurofibromas |
| R | NFRxUnsure | not sure how neurofibromas treated |
| S | NFRxOther | neurofibromas treated with other therapy |
| T | NFRxOtherVal | specify other therapy neurofibromas treated |
| U | SpinalNFbyMRI | diagnosed by MRI neurofibromas on or near spine |
| V | SpinalNFRxDrug | paraspinal neurofibromas treated with drugs |
| W | SpinalNFRxRadiation | paraspinal neurofibromas treated with radiation |
| X | SpinalNFRxSurgery | paraspinal neurofibromas treated with surgery |
| Y | SpinalNFRxNone | no treatment of paraspinal neurofibromas |
| Z | SpinalNFRxUnsure | not sure how paraspinal neurofibromas treated |
| AA | SpinalNFRxOther | paraspinal neurofibromas treated other therapy |
| AB | SpinalNFRxOtherVal | specify other treatment paraspinal neurofibromas |
| AC | PlexiNF | have plexiform neurofibromas and if so how many |
| AD | PlexiNFHead | plexiform in head area |
| AE | PlexiNFNeck | plexiform in neck area |
| AF | PlexiNFChest | plexiform in chest area |
| AG | PlexiNFArms | plexiform in arm(s) |
| AH | PlexiNFLegs | plexiform in leg(s) |
| AI | PlexiNFAbd | plexiform in abdomen |
| AJ | PlexiNFUnsure | not sure of plexiform location |
| AK | PlexiNFSpine | plexiform on spine |
| AL | PlexiNFBack | plexiform on back |
| AM | PlexiNFOther | plexiform in other area |
| AN | PlexiNFOtherVal | specify with other location plexiform |
| AO | PlexiNFRxDrug | plexiform treated with drugs |
| AP | PlexiNFRxRadiation | plexiform treated with radiation |
| AQ | PlexiNFRxSurgery | plexiform treated with surgery |
| AR | PlexiNFRxUntreated | no treatment of plexiform |
| AS | PlexiNFRxUnsure | not sure how plexiform treated |
| AT | PlexiNFRxOther | plexiform treated with other therapy |
| AU | PlexiNFRxOtherVal | specify with what other therapy plexiform treated |
| AV | OpticGlioma | diagnosed with optic nerve pathway glioma |
| AW | OGRxDrug | optic nerve pathway glioma treated with drugs |
| AX | OGRxRadiation | optic nerve pathway glioma treated with radiation |
| AY | OGRxSurgery | optic nerve pathway glioma treated with surgery |
| AZ | OGRxUntreated | no treatment of optic nerve pathway glioma |
| BA | OGRxUnsure | not sure how optic nerve pathway glioma treated |
| BB | OGRxOther | optic nerve pathway glioma treated other therapy |
| BC | OGRxOtherVal | specify other therapy optic nerve pathway glioma |
| BD | PeriphNerveSheathTumor | malignant peripheral nerve sheath tumor (MPNST) |
| BE | MPNSTRxDrug | MPNST treated with drugs |
| BF | MPNSTRxRadiation | MPNST treated with radiation |
| BG | MPNSTRxSurgery | MPNST treated with surgery |
| BH | MPSTRxUntreated | no treatment of MPNST |
| BI | MPSTRxUnsure | not sure how MPNST treated |
| BJ | MPSTRxOther | MPNST treated with other therapy |
| BK | MPSTRxOtherVal | specify with what other therapy MPNST treated |
| BL | EverClincalTrial | participated in clinical trial for any tumor |
| BM | EverBoneFx | have or ever had bone fractures |
| BN | EverOsteoporosis | have or ever had osteoporosis |
| BO | EverSphenoidDysplasia | have or ever had sphenoid dysplasia |
| BP | EverScoliosis | have or ever had scoliosis |
| BQ | ScolioRxBrace | scoliosis treated with brace |
| BR | ScolioRxRods | scoliosis treated by fixation with rods |
| BS | ScolioRxDrugs | scoliosis treated with drugs |
| BT | ScolioRxUntreated | scoliosis not treated |
| BU | ScolioRxUnsure | unsure how scoliosis treated |
| BV | ScolioRxOther | scoliosis treated with other therapy |
| BW | ScolioRxOtherVal | specify with what other therapy scoliosis treated |
| BX | BoneBowing | bowing of long bones of lower leg or forearm |
| BY | BoneBowRxAmp | bone bowing treated with amputation |
| BZ | BoneBowRxCast | bone bowing treated with casting |
| CA | BoneBowRxDrug | bone bowing treated with drugs |
| CB | BoneBowRxSurgery | bone bowing treated with surgery |
| CC | BoneBowRxUntreated | bone bowing not treated |
| CD | BoneBowRxUnsure | unsure how bone bowing treated |
| CE | BoneBowRxBrace | bone bowing treated with bracing |
| CF | BoneBowRxOther | bone bowing treated with other therapy |
| CG | BoneBowRxOtherVal | specify other therapy bone bowing treated |
| CH | LearningDifficulties | have or ever had learning difficulties in school |
| CI | LearningRxTutor | had extra tutoring |
| CJ | LearningRxDrug | had ADHD medication |
| CK | LearningLovastatTrial | participation in lovastatin trial |
| CL | LearningRxNone | no treatment for learning difficulties |
| CM | LearningRxUnsure | not sure how learning difficulties treated |
| CN | Itching | ever suffered from itching |
| CO | HCHighBP   have or ever had high blood pressure | have or ever had high blood pressure |
| CP | HCBrain   have or ever had | have or ever had brain issues such as stroke |
| CQ | HCHeart | have or ever had heart problems |
| CR | HCKidney | have or ever had kidney problems |
| CS | HCGlomusTumor | have or ever had glomus tumors |
| CT | HCHeadache | have or ever had headache (migraine) |
| CU | HCHydroceph | have or ever had hydrocephalus |
| CV | HCCancer | have or ever had cancer |
| CW | HCSeizures | have or ever had seizures |
| CX | HCEarlyPuberty | have or had early puberty |
| CY | HCLatePuberty | have or had late puberty |
| CZ | HCPoorWtGain | have or ever had poor weight gain |
| DA | HCEndocrineInPregContraCept | hormone imbalance in pregnancy or on contraceptive |
| DB | NoHealthConcerns | none of the above health problems |
| DC | HealthConcernUnsure | not sure if ever had any of above health problems |
| DD | Health Concern Other |  |
| DE | NFRelatedPain | have pain and if so ever prescribed painmedication |
| DF | FemaleNubile | female of childbearing age |
| DG | FemaleBreastCa | ever diagnosed with breast cancer |
| DH | BreastCaRxDrug | breast cancer treated with drugs |
| DI | BreastCaRxMastect | breast cancer treated with mastectomy |
| DJ | BreastCaRxRadiation | breast cancer treated with radiation |
| DK | BreastCaUntreated | breast cancer not treated |
| DL | BreastCaRxNotApplic | breast cancer treatment not applicable |
| DM | BreastCaRxLumpect | breast cancer treated with lumpectomy |
| DN | BreastCaRxOther | breast cancer had other treatment |
| DO | BreastCaRxOtherVal | specify other treatment for breast cancer |
| DP | EverPregnant | ever pregnant |
| DQ | NFNumIncrInPreg | in pregnancy number of neurofibromas increased |
| DR | NFNumUnchangedInPreg | in pregnancy number of neurofibromas unchanged |
| DS | NFSizeIncrInPreg | in pregnancy size of neurofibromas increased |
| DT | NFSizeUnchangedInPreg | in pregnancy size of neurofibromas unchanged |
| DU | NFUnchangedInPreg | if ever pregnant, no change in neurofibromas |
| DV | ChangeInPainInPreg | in pregnancy size of pain become more severe |
| DW | OBComplicationsInPreg | in pregnancy any obstetrical complications |
| DX | OBComplicationsOtherVal | specify obstetrical complications |
| DY | OtherHealthProblems | any other health problems, describe |

**NF2**

| A | PatientID | unique patient identifier |
| --- | --- | --- |
| B | FamilyID | unique family identifier |
| C | SessionID | unique session identifier |
| D | ClinicalStatus | did patient meet diagnostic criteria for NF2 |
| E | RelationToCase | is data self-reported or reported by parent/guardian |
| F | Syndrome | type of NF |
| G | Gender | male or female |
| H | DxByHCP | Were you diagnosed by a health care provider? |
| I | FirstSx | First symptom associated with your NF2 |
| J | FirstSxOtherVal | First symptom if not listed as choice in above question |
| K | TwoOrMoreTumors | two or more individual tumors |
| L | VestibSchwannoma | vestibular schwannoma |
| M | Meningioma | meningioma |
| N | Ependymoma | ependymoma |
| O | SpinalSchwannoma | spinal schwannoma |
| P | OtherSchwannoma | other schwannoma |
| Q | SchwannomaUnsure | not sure if had other schwannoma |
| R | Neurofibroma | neurofibroma |
| S | Glioma | glioma |
| T | NFTumorOther | other NF tumor |
| U | NFTumorOtherVal | specify other type of NF tumor |
| V | Cataract | juvenile cataract |
| W | HearingLossLeft | rate level of hearing loss in left ear |
| X | HearingLossRight | rate level of hearing loss in left ear |
| Y | Imbalance | rate level of dizziness/imbalance |
| Z | Tinnitus | rate level of tinnitus |
| AA | Facial Weakness | rate level of facial weakness |
| AB | NFRelatedPain | rate level of pain associated with your NF2 |
| AC | Vision | rate level of vision change/loss associated with your NF2 |
| AD | BiggestNFProblem | your biggest problem from NF2 |
| AE | BiggestNFProblemOtherVal | if other, specify |
| AF | SurgeriesForVestSchwan | number of surgeries to treat vestibular schwannoma |
| AG | SurgeriesForMeningioma | number of surgeries to treat meningioma |
| AH | SurgeriesForPeriphSchwan | number of surgeries to treat peripheral schwannoma |
| AI | RadiationCoursesIntracranial | courses radiation/radiosurgery for an intracranial tumor |
| AJ | AnyTumorBeingWatched | tumors doctors have chosen to observe rather than treat |
| AK | WatchMeningioma | watching meningioma |
| AL | WatchEpendymomo | watching ependymoma |
| AM | WatchSpinalSchwan | watching spinal schwannoma |
| AN | WatchOthSchwann | watching other schwannoma |
| AO | WatchSpinalMeningioma | watching spinal schwannoma |
| AP | WatchTumorUnsure | not sure if watching any tumor |
| AQ | WatchTumorOther | watching a different tumor than those listed |
| AR | WatchTumorOtherVa | specify other tumor watched |
| AS | CochlearImplant | implanted with a cochlear implant |
| AT | CochlearImplantBenefit | does the cochlear implant help you |
| AU | AuditoryBrainImplant | implanted with an auditory brainstem implant |
| AV | AuditoryBrainImplantBenefit | does the auditory brainstem implant help you |
| AW | TrialMed | taken trial medication specifically for NF2 |
| AX | Lapatinib | taken lapatinib |
| AY | Gelfitinib | taken gelfitinib |
| AZ | Erlotinib | taken erlotinib |
| BA | Bevacizumab | taken bevacizumab |
| BB | Imatinib | taken imatinib |
| BC | PTC299 | taken PTC299 |
| BD | SOM230B | taken SOM230B |
| BE | TrialMedOther | taken other trial medication |
| BF | TrialMedOtherVal | specify other trial medication |
| BG | OTCAnySupplement | taken over-the-counter supplements to treat tumors |
| BH | OTCBio30 | taken over the counter Bio30 |
| BI | OTCNacetylCysteine | taken over the counter N-acetylcysteine |
| BJ | OTCGlutathione | taken over the counter Glutathione |
| BK | OTCWhet | taken over the counter whey protein |
| BL | OTCArtepillin | taken over the counter Artepillin C |
| BM | OTCBioperine | taken over the counter Bioperine |
| BN | OTCBlackSeedOil | taken over the counter Black Seed Oil |
| BO | OTCCoenzymeQ10 | taken over the counter Coenzyme Q10 |
| BP | OTCCurcumin | taken over the counter Curcumin from turmeric |
| BQ | OTCGreenTea | taken over the counter EGCG from green tea |
| BR | OTCBroccoli | taken over the counter isothiocyanate sulforaphane from broccoli |
| BS | OTCBoswelia | taken over the counter 5-loxin from Boswellia |
| BT | OTCOmega3 | taken over the counter omega-3 resveratrol |
| BU | OTCVitaminC | taken over the counter Vitamin C |
| BV | OTCVitaminD | taken over the counter Vitamin D |
| BW | OTCVitaminE | taken over the counter Vitamin E |
| BX | OTCOther | taken other over the counter supplement |
| BY | OTCOtherValue | specify other over the counter supplement |

**Schwannomatosis**

| A | NF1RowNo | row number of patient |
| --- | --- | --- |
| B | PatientID | unique patient identifier |
| C | FamilyID | unique family identifier |
| D | SessionID | unique session identifier |
| E | ClinicalStatus | meets diagnostic criteria for schwannomatosis |
| F | RelationToCase | data self-reported or by parent/guardian |
| G | Syndrome | type of NF |
| H | Gender | male or female |
| I | DxbyHCP | were you diagnosed by a health care provider? |
| J | TwoOrMoreTumors | have more than one schwannoma |
| K | BrainMRI | vestibular schwannoma ruled out by MRI |
| L | VestibSchwannomma | have vestibular schwannoma |
| M | Pain | have pain |
| N | PainSeverity | severity of pain |
